# Delta-24-RGD combined with radiotherapy exerts a potent antitumor effect in diffuse intrinsic pontine glioma and pediatric high grade glioma models

**DOI:** 10.1186/s40478-019-0714-6

**Published:** 2019-04-29

**Authors:** Naiara Martinez-Velez, Miguel Marigil, Marc García-Moure, Marisol Gonzalez-Huarriz, Jose Javier Aristu, Luis-Isaac Ramos-García, Sonia Tejada, Ricardo Díez-Valle, Ana Patiño-García, Oren J. Becher, Candelaria Gomez-Manzano, Juan Fueyo, Marta M. Alonso

**Affiliations:** 1 The Health Research Institute of Navarra (IDISNA), Pamplona, Navarra Spain; 2 Program in Solid Tumors, Foundation for the Applied Medical Research, Pamplona, Navarra Spain; 3 Department of Pediatrics, Clínica Universidad de Navarra, University of Navarra, CIMA Building, Avd. Pio XII, 55, Pamplona, Spain; 4 Division of Neurosurgery, Lariboisière University Hospital, 2 Rue Ambroise Paré, 75475 Paris, cedex 10 France; 5 Department of Radiation Oncology, Clínica Universidad de Navarra, University of Navarra, Pamplona, Spain; 6 Department of Neurosurgery, Clínica Universidad de Navarra, University of Navarra, Pamplona, Spain; 7 Department of Pediatrics, Northwestern University and Division of Pediatric Hematology-Oncology and Stem Cell Transplant, Ann & Robert H. Lurie Children’s Hospital, Chicago, IL USA; 8 Department of Genetics, The University of Texas MD Anderson Cancer Center, Houston, TX USA; 9 Department of NeuroOncology, The University of Texas MD Anderson Cancer Center, Houston, TX USA; 10 Department of Neurosurgery, The University of Texas MD Anderson Cancer Center, Houston, TX USA

**Keywords:** pHGG, DIPG, Radiotherapy, Oncolytic virus, DNA damage, Immune response

## Abstract

**Abstract:**

Pediatric high grade gliomas (pHGG), including diffuse intrinsic pontine gliomas (DIPGs), are aggressive tumors with a dismal outcome. Radiotherapy (RT) is part of the standard of care of these tumors; however, radiotherapy only leads to a transient clinical improvement. Delta-24-RGD is a genetically engineered tumor-selective adenovirus that has shown safety and clinical efficacy in adults with recurrent gliomas. In this work, we evaluated the feasibility, safety and therapeutic efficacy of Delta-24-RGD in combination with radiotherapy in pHGGs and DIPGs models. Our results showed that the combination of Delta-24-RGD with radiotherapy was feasible and resulted in a synergistic anti-glioma effect in vitro and in vivo in pHGG and DIPG models. Interestingly, Delta-24-RGD treatment led to the downregulation of relevant DNA damage repair proteins, further sensitizing tumors cells to the effect of radiotherapy. Additionally, Delta-24-RGD/radiotherapy treatment significantly increased the trafficking of immune cells (CD3, CD4+ and CD8+) to the tumor niche compared with single treatments.

In summary, administration of the Delta-24-RGD/radiotherapy combination to pHGG and DIPG models is safe and significantly increases the overall survival of mice bearing these tumors. Our data offer a rationale for the combination Delta-24-RGD/radiotherapy as a therapeutic option for children with these tumors.

**Significance:**

Delta-24-RGD/radiotherapy administration is safe and significantly increases the survival of treated mice. These positive data underscore the urge to translate this approach to the clinical treatment of children with pHGG and DIPGs.

**Electronic supplementary material:**

The online version of this article (10.1186/s40478-019-0714-6) contains supplementary material, which is available to authorized users.

## Introduction

Pediatric high grade gliomas (pHGG) and diffuse intrinsic pontine gliomas (DIPGs) are malignant tumors present with an aggressive behavior [7]. Integrated molecular profiling has contributed to renew the classification of these tumors by considering the mutations encoding histone H3 variants that determine localization, age of presentation, clinical outcome or even radiological features [18, 25, 30, 41].

The current standard therapy for pHGG consists of maximal surgical resection followed by temozolomide/radiotherapy (RT) [35]. In the case of DIPGs, effective therapeutic options are limited, and the standard of care is RT. RT offers a temporal decrease of clinical symptoms and an increase in the overall survival; however, it is not curative [12] . Despite combined efforts to develop new therapies for these aggressive tumors, over the last decade, the overall survival is 15 months for pHGG patients and approximately 9 to 11 months for DIPGs [16].

RT induces DNA damage [24], resulting in the triggering of either cell death programs or cell survival mechanisms, such as apoptosis, necrosis or autophagy, among others [6]. Radiation-induced cell responses mediated by DNA damage cause tumor antigens release, generation of ROS species or production of cytokines, which awake the immune system [31]. Recent studies demonstrate that RT also induces an immunogenic cell death that promotes the recruitment of different immune populations to the tumor bed. In some instances, RT triggers an abscopal effect which results in an effective immunity against the tumor [9]. However, the RT mediated abscopal effect is seen in few patients and many times is hampered by the tolerance and an immunosuppressive tumor microenvironment [28].

In this study, we evaluated whether the combination of the oncolytic adenovirus, Delta-24-RGD (DNX-2401) [36], genetically engineered to destroy cancer cells, in combination with RT would result in a superior antitumor effect in pHGG and DIPGs when compared to either agent alone. Delta-24-RGD administration has been demonstrated to be safe and therapeutically effective in a subset of adult patients with recurrent glioblastoma [22]. Moreover, clinical and preclinical studies with Delta-24-RGD have shown that part of the antitumor effect is due to the capacity of the virus to boost or awake the patient’s immune system [15]. We have previously shown that administration of Delta-24-RGD alone resulted in a robust antitumor effect in vitro and in vivo in pHGGs and DIPGs models (Martinez-Velez et al., 2019, *nature communication*, *in press*). Moreover, our group and others have shown that adenoviral infection inhibits the cellular DNA repair machinery to increase its replication potency [34]. We hypothesized that DNA repair inhibition by viral administration could sensitize tumor cells to irradiation, increasing the therapeutic effect [29]. Furthermore, RT/Delta-24-RGD administration will increase the release of tumor antigens overcoming the “cold” status of pHGG and DIPG tumors and triggering a stronger immune response that could translate into a synergistic antitumor effect and an increase of the overall survival in these patients.

## Material and methods

### Cell lines and culture conditions

Pediatric glioma CHLA-03-AA (H3 WT) was obtained from the America Type Culture Collection (ATCC, Manassas, VA). PBT-24 pediatric glioma cell line was developed from a biopsy (H3 WT) obtained at the University Clinic of Navarra from a 13-year-old boy. Tumor samples were obtained with a signed-informed consent. Tumors were cut into smaller pieces, and cells were dissociated enzymatically. Cell obtained from dissociation were cultured with RPMI medium supplemented with 10% FBS and 1% antibiotic. Cell lines obtained from ATCC or Chilren Oncology Group (COG) were cultured following manufacturer specifications. The DIPG cell line TP54 (H3.3K27M) was kindly provided by Drs. Marie-Pierre Junier and Hervé Cheneiwess (INSERM Institute, Paris, France), and the SU-DIPG IV (H3.1K27M) cell line was a kind gift from Michelle Monje (Stanford University, CA). The DIPG cell line was maintained as neurospheres cultured in a specialized serum-free basal medium complemented with a human neural stem cell proliferation supplement (NeuroCult™ NS-A Proliferation Kit, #05751, STEMCELL Technologies) supplemented with basic fibroblast growth factor and epidermal growth factor (20 ng/mL Sigma-Aldrich, St Louis, MO). All cells were maintained in a humidified atmosphere containing 5% CO_2_ at 37 °C. All of the cell lines were tested and authenticated at the CIMA Genomic Core Facility (Pamplona, Spain) using short tandem repeats DNA profiling.

The murine DIPG cell lines NP53 and XFM were provided by Dr. Becher (Northwestern University, Chicago, IL). Cell lines were generated from DIPG tumors arisen in genetically modified mice. The NP53 cell line was generated from tumors that arose in a DIPG mouse model induced by PDGF-B signaling, p53 loss, and ectopic H3.3-K27 M [10]. The XFM cell line was generated from tumors developed in a mouse model driven by PDGF-B signaling and Ink4a and ARF loss [3].

### Adenovirus construction and infection

Construction of Delta-24-RGD and viral infection have been previously described [26, 40].

### Viral replication assays

pHGG and DIPG cells were seeded at a density of 2 × 10^5^ cells/well in 6-well plates and infected with 10 MOI of Delta-24-RGD, and 4 h after infection, these cells were irradiated with three different doses (3, 6 and 12 Gy). Three days later, cells were collected, and the final amount of virus was determined by means of a method based on anti-hexon staining in HEK293 cells [4].

### Cell viability assay

Cells were seeded at a density of 2 × 10^5^ cells per well in 6-well plates, and the next day, cells were infected with 10 MOIs of Delta-24-RGD. In addition, cells were irradiated at doses of 3, 6 and 12 Gy. Cell viability was assessed 5 days later using trypan blue. Dose–response curves were analyzed using GraphPad software. Quantification of viability was measured in triplicate, and each experiment was performed three times. After fitting the combined dose-response curve from a single representative experiment to a Chou–Talalay line, Chou–Talalay combination indices (CIs) were calculated with Calcusyn software (Biosoft, Cambridge, UK). Levels of interaction are defined as follows: CI > 1.1 indicates antagonism, CI between 0.9 and 1.1 indicates additive effect, and CI < 0.9 indicates synergy [5]. A mean CI was calculated from data points with the fraction affected (FA) > 0.5. The FA range used to calculate the average CI values in the combination experiments did not include CI values of FA < 0.5, which was not considered a relevant growth inhibition because one aims to achieve the maximal effect of the combination tested on cancer cells.

### Immunoblotting

For immunoblotting assays, samples were subjected to SDS-Tris-glycine gel electrophoresis. Membranes were incubated with the following antibodies: E1A, (Santa Cruz Biotechnology, Santa Cruz, CA), fiber (NeoMarkers, Fremont, CA), Mre-11, Rad 50, Rad 51, pH2Ax, MPG (Cell Signaling, Danvers, MA) and GRB-2 (Sigma-Aldrich). The membranes were developed according to Amersham’s enhanced chemiluminescence protocol.

### Animal studies

Ethical approval for animal studies was granted by the Animal Ethical Committee of the University of Navarra (CEEA; Comité Etico de Experimentación Animal under the protocol number CEEA/069–13). All animal studies were performed in the veterinary facilities of the Center for Applied Medical Research in accordance with institutional, regional, and national laws and ethical guidelines for experimental animal care. For the orthotopic supratentorial model, CHLA-03-AA cells (5 × 10^5^) were engrafted by injection into the caudate nucleus of athymic mice. TP54 cells (5 × 10^5^) developed DIPG tumors by injection of those cells into the pons of athymic mice in both models, we have utilized a guide-screw system. (Taconic Farms, Inc.). NP53 cells (5 × 10^5^) were implanted in transgenic mice, kindly provided by Dr. Oren Becher. Cells were administered in 3–4 μl of PBS. Animals were randomized to 2 or 4 groups (controls without treatment, Delta-24-RGD, irradiated, and combination of radiation and Delta-24-RGD). Delta-24-RGD (10^7^ pfu/animal) was administered intracranially once in 3–4 μl 3 days after cell implantation. A week later, brain tumors were irradiated (4 Gy) using the bolt as a guide to administer irradiation to that area.

### Tumor establishment procedure

Under aseptic conditions and with all materials sterilized according to standard techniques, mice of 4 weeks of age were anesthetized by intraperitoneal injection with ketamine and xilacyne solution. The animal heads were supported by a couple of rolled gauzes so that when the screw was inserted, pressure applied over neck and head structures was better tolerated by the animal.

We prepared mice head skin with povidone iodine solution prior to make a 5 mm-long lineal skin incision with 23-size scalpel and expose skull sutures. We first made a small mark according to the coordinates with a small 15-gauge needle which was subsequently widened with a hand-controlled twist drill which penetrates the skull. Next, we introduced the screw with its specific screwdriver by applying slight pressure throughout the previous twist hole. The coordinates for generation of DIPG tumors are 1.0 mm right to lambda and just posterior (0.8 mm) to lambdoid suture, while coordinates for pHGG tumors are from bregma (intersection between coronal and sagittal suture) 1 mm anterior and 2.5 mm to the right. Thereafter the needle of Hamilton syringe is slowly introduced into the hole by applying gentle pressure until the sleeve/cuff from the syringe reaches the screw surface. The desired depth to reach brainstem is 6.5 mm and depth for hemispheric tumors (pHGG) is 3.5 mm. Cell suspension was carefully injected using an infusion pump (Harvard Apparatus) over 20 min.

### Comet assay

Cell lines were irradiated with ascending doses (ranging from 3 to 12 Gy) and infected with Delta-24-RGD at 10 MOIs; 72 h later, cells were recollected. Cells were prepared following the manufacturer protocol provided by CometAssay® kit (Trevigen, Inc., Gaithersburg, MD).

### Immunohistochemical analysis

The paraffin-embedded sections of the mice brains were immunostained for antibodies specific for adenoviral mouse-hexon (Chemicon International, Inc., Temecula, CA), adenovirus rabbit-E1A, (Santa Cruz Biotechnology, Santa Cruz, CA), pH2Ax (Cell Signaling, Danvers, MA), CD3 (NeoMarkers, Fremont, CA), CD4 (Abcam, Cambridge, MA) CD8a (Cell Signaling, Danvers, MA), FoxP3 (eBiosciences, Thermo Fisher, Waltham, MA) and vimentin clone V9 (IS30, Dako Denmark A/S, Glostrup, Denmark), following manufacturer procedures. For immunohistochemical staining, Vectastain ABC kits (Vector Laboratories Inc., Burlingame, CA) were used according the manufacturer’s instructions.

### Statistical analysis

For the in vitro experiments, data are expressed as the mean ± SD, and comparisons were evaluated by the two-tailed Student’s *t* test or ANOVA. The effect of Delta-24-RGD and RT, alone or in combination, and pHGG and DIPG xenografts was assessed by plotting survival curves according to the Kaplan-Meier method. Survival in different treatment groups was compared using the log-rank test. The program GraphPad Prism 5 (Statistical Software for Sciences) was used for the statistical analysis.

## Results

### Combination of Delta-24-RGD with RT exerts a synergistic antitumor effect in pHGG and DIPG in vitro and in vivo

**Fig. 1:**
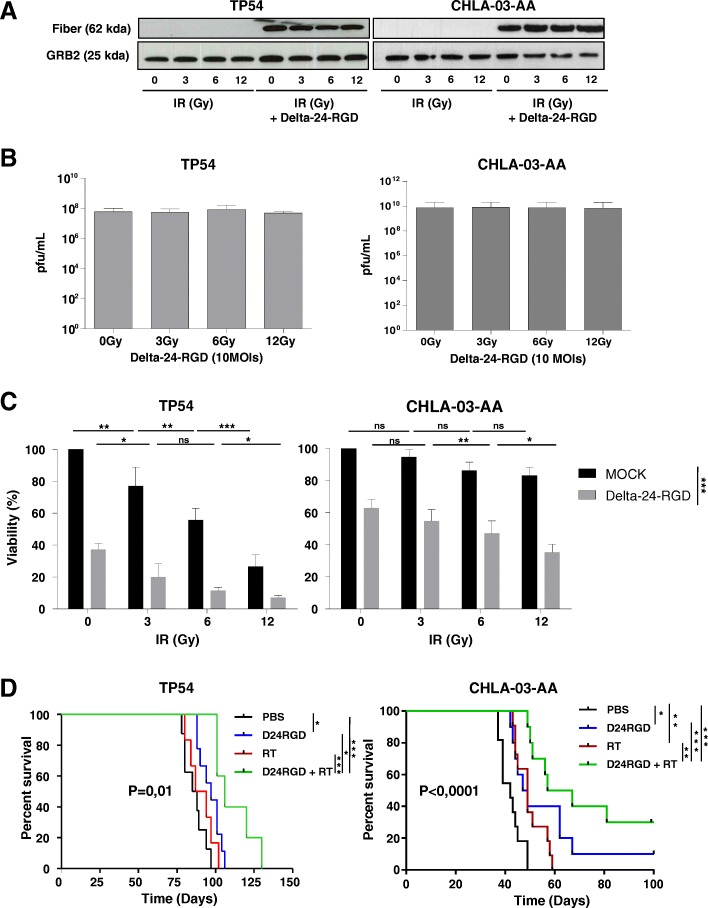
Radiotherapy is amenable to combine with Delta-24- in vitro *and* in vivo in the DIPG and pHGG models. **a** Evaluation by western blotting of the expression of viral proteins after Delta-24-RGD (10 MOIs) infection and subsequent irradiation (3 , 6 and 12 Gy) in TP54 and CHLA-03-AA. **b** Quantification of Delta-24-RGD replication in the indicated cell lines irradiated with different Gy doses. The viral titers were determined 3 days after infection at an MOI of 10 by an anti-hexon staining-based method in 293 cells and expressed as plaque-forming units (pfu) per milliliter. Data are shown as the mean ± SD of three independent experiments and analyzed with two-tailed Student-t test, not significant differences were found. **c** Cell viability analyses of irradiated cells at the indicated Gy doses alone (Mock; control with a mock infection) or in combination with Delta-24-RGD. Cell viability was assessed 5 days after irradiation and viral infection using an automatic cell counter that measures cell viability (life, death and total cells) with the standard trypan blue reaction. Data are shown as the percentage (the mean ± SD) of viability after treatments and relative to control cells (neither infected nor irradiated). Statistical significance were calculated using two-tailed Student-t test, ns, not significative; *, *P* < 0.05; **, *P* < 0.01; ***, *P* < 0.001. **d** Kaplan-Meier survival curves of nude mice bearing orthotopic DIPGs (TP54) or pHGG (CHLA-03-AA) tumors that were treated with either Delta-24-RGD (10^7^ pfu), irradiation (4 Gys) or combined treatment. Log-rank test were used for statistical analyses

First, to evaluate whether irradiation would interfere with viral replication, we infected pHGG and DIPG cells with Delta-24-RGD (10 MOIs) followed by increasing doses of RT: 3 , 6 and 12 Gy. After the combined treatment, we observed a robust expression of the viral late protein fiber regardless of the RT dosage used (Fig. 1a and Additional file 1: Figure S1A). This result suggested that RT does not interfere with the viral cycle. To support this notion, we quantified the viral progeny present in cells after irradiation with increasing Gys. We found that Delta-24-RGD replication was not hindered by any of the irradiation doses evaluated (Fig. 1b and Additional file 1: Figure S1B). These data confirmed the feasibility of combining RT with the Delta-24-RGD virus. Next, we evaluated the anticancer effect of this combination in a panel of the pHGG and DIPG cell lines. Our results showed that RT alone, at the highest dose used of 12 Gy, induced only a modest increment of cell death, 30–40%, in the pHGG (CHLA-03-AA and PBT-24) and DIPG cell lines (TP54 and SU-DIPG IV) (Fig. 1c, Additional file 1: Figure S1C and Table 1). The TP54 DIPG cell line was more susceptible to RT, with a 70% cell death at the 12 Gy dose (Fig. 1c and Additional file 1: Figure S2A).

**Table 1:** Median-effect doses (%viability) of Delta-24-RGD alone or combined with different doses of radiotherapy in the pHGG and DIPG cell lines. The value is the viability percentage ± SD of cells irradiated with different doses or cells irradiated with different doses and infected with a single viral dose. The interaction between RT and Delta-24-RGD in pHGG and DIPG cell lines was measured by combination index (CI) values. The interaction was measured according to the combination index values. Combination index values > 1.3 indicated antagonism, values between 1.1 and 1.3 indicated moderate antagonism, values between 0.9 and 1.1 indicated additivity, values between 0.8 and 0.9 indicated slight synergy, values between 0.6 and 0.8 indicated moderate synergy, values between 0.4 and 0.6 indicated synergy, and values < 0.4 indicated strong synergy. Each combination was studied in three independent experiments, the differences of which were not statistically significant. The results of single experiments are shown

	Cell lines	IR (Gy)	Mock	+Delta-24-RGD	CI
pHGG	CHLA-03-AA	3	94.76 ± 7.07	54.77 ± 7.07	1.10
		6	86.26 ± 5.2	47.17 ± 7.74	0.76
		12	53.10 ± 5.23	35.33 ± 5.03	0.41
	PBT-24	3	90.1 ± 3.03	38.2 ± 3.13	0.45
		6	81.4 ± 6.74	29.9 ± 7.22	0.36
		12	73.9 ± 4.46	19.9 ± 8.66	0.19
DIPG	TP54	3	93.5 ± 9.19	16.0 ± 7.07	0.33
		6	58.5 ± 7.7	10.2 ± 0.35	0.18
		12	22.5 ± 0.7	6.5 ± 0.7	0.17
	SU-DIPG IV	3	88.0 ± 1	38.2 ± 3.13	0.97
		6	82.0 ± 6.74	29.9 ± 7.22	0.48
		12	73.9 ± 4.46	19.9 ± 8.66	0.19

*IR* Irradiated dosage, *CI* Combinatory index, *Mock* non infected cells

Combination of RT with Delta-24-RGD induced an increase in the cytotoxicity with a viability decrease of approximately 20–40% when compared with the single treatment (Fig. 1c and Table 1) (*P* < 0.001). Evaluation of the combinatory index (CI) showed that RT plus Delta-24-RGD had a synergistic antiglioma effect in all doses tested in the PBT-24 and TP54 cell line. In the CHLA-03-AA and SU-DIPG IV cell lines, combination treatment presented a synergistic effect (CI = 0.17–0.76) at the highest irradiation doses (6 and 12 Gy); meanwhile, at the lowest irradiation dose (3 Gy), an additive effect with Delta-24-RGD resulted (CI = 0.9–1.1) (Table 1).

Next, we wanted to assess whether in vivo combination of Delta-24-RGD with RT would show the same synergistic effect that we observed in vitro in the pHGG and DIPG cell lines to determine the optimal dose to the mice pons. The results showed that any of tested doses evaluated presented toxicity (Additional file 1: Table S1). Next, we evaluated which dose accurately reproduced the clinical behavior presented in patients. We established that 4 Gy irradiation produces a transient delay in tumor growth similar to that in patients but with no curative effect.

We used the CHLA-03-AA (pHGG) and TP54 (DIPG) cell lines to assess the in vivo combination efficacy. A single dose (10^7^ pfu) of Delta-24-RGD was injected intratumorally at day 3 followed by RT (4 Gy) at day 4. Animals were monitored during the experiment, and they were sacrificed when they presented signals of physical deterioration derived from high tumor burden presence.

Survival analyses showed that RT increased the median survival of mice in 6 days in the TP54 model (*P* = 0.39) (Fig. 1d and Table 2) and in 7 days in the CHLA-03-AA model (*P* = 0.003) (Fig. 1d). Combination of virus/RT compared with single RT significantly increased the survival in both models (*P* = 0.009 and *P* < 0.006, respectively). Importantly, combination treatment of mice bearing the CHLA-03-AA resulted in 3 long-term survivors free of disease (*N* = 3) (*P* < 0.0001). Mice bearing the TP54 orthotopic DIPG model also benefitted from the RT/virus combination with a significant increase of 20 days (*P = 0.01)* in the overall survival.

**Table 2:** Median survival and log-rank test *P*-value of different treated groups in a DIPG and pHGG model. *P* value 1 shows the *P* value of the comparison of each group with the PBS group, *P* value 2 compares each group with the Delta-24-RGD-treated group and *P* value 3 compares radiotherapy with the combination group

			Survival (Days)	*P* value [7]	*P* value [18]	*P* value [30]
DIPG	TP54	Control	83			
		Delta-24-RGD	95	0,04		
		RT	89	0,39	0,2	
		RT + Delta-24-RGD	106	0,005	0,02	0,009
pHGG	CHLA-03-AA	Control	42			
		Delta-24-RGD	48	0,01		
		RT	49	0,003	0,2	
		RT + Delta-24-RGD	62	< 0,0001	0,1	0,006

These data suggest that combination of virus and RT could be synergistic not only in vitro but also importantly in vivo, allowing a reduction in the RT doses to achieve the same effect.

In summary, the combination of irradiation and Delta-24-RGD is a feasible therapeutic strategy that increases the antitumor effect in vitro and increases the overall survival when compared with single treatment (RT or Delta-24-RGD) administration in pHGG and DIPG models.

### Delta-24-RGD inhibits important proteins involved in the DNA damage cellular response

RT induces DNA damage in cancer cells, and hence, if left unrepaired, the cells die [24]. Of importance, our group and others [14, 17, 20, 34] have shown that adenoviral proteins are able to inhibit different components of the cell DNA damage repair machinery to facilitate their replication in the host. Interestingly, viral inhibition of the DNA damage repair protein could contribute to sensitizing tumor cells to agents that cause DNA damage. Therefore, we evaluated the expression of several proteins involved in DNA damage to understand whether the inhibition of the DNA damage repair machinery could be underlying the synergistic effect of treatment combination.

**Fig. 2:**
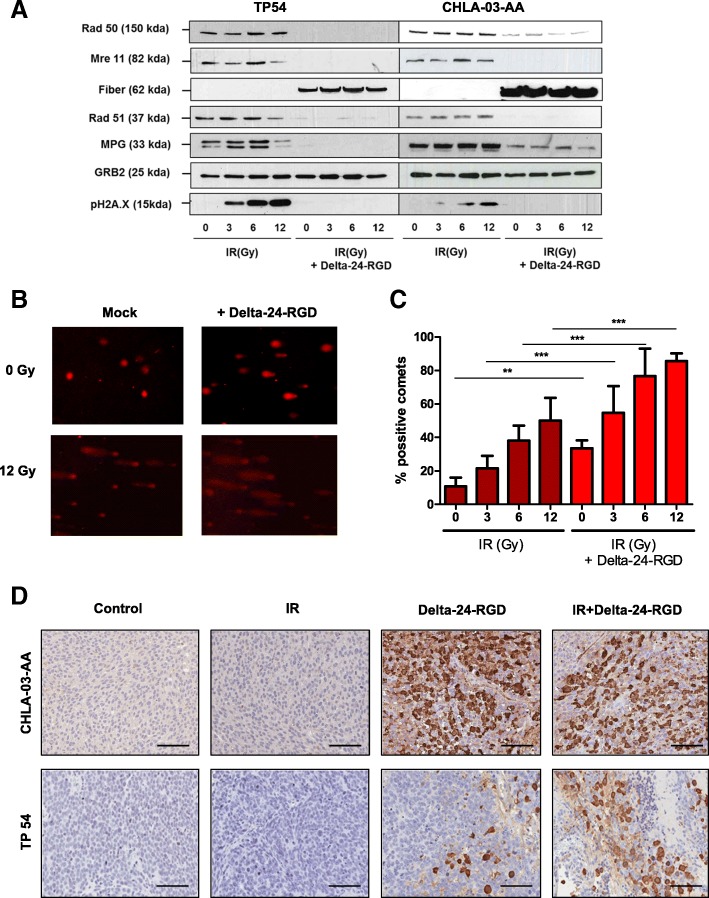
Delta-24-RGD downregulation of the cellular DNA damage repair machinery in the DIPG and pHGG cell lines. **a** Expression analyses by western blotting of the relevant proteins involved in the DNA damage response to RT in the DIPG and pHGG cell lines after the indicated treatments. The proteins levels were evaluated 72 h after cells were treated. **b** Evaluation of DNA damage upon treatment with Delta-24-RGD and/or RT by the comet assay. TP54 cells were administered the indicated treatments, and 72 h later, the induction of comets was assessed. Representative photomicrographs of comets shown by the cells after the indicated treatment (magnification, × 200). **c** Quantification of positive cells showing comets after the indicated treatment. Data are shown as the percentage of comet tails found per treatment percentage (*n* = 500 cells per treatment); bars represent means ± SD. All experiments were performed in triplicate and analyzed using two-way ANOVA and corrected for multiple comparision with Bonferroni posttest; **, *P* < 0.01 and ***, *P* < 0.001. **d** Hexon immune-staining representative images (scale bar =100um) after the indicated treatments. The above images images show differences in hexon protein expression in CHLA-03-AA tumors while below images show hexon staining in TP54 tumors

We observed that Delta-24-RGD infection resulted in the inhibition of cellular machinery, involving Rad50 and Mre11 proteins; both proteins are part of the MRN complex [21, 33] which is involved in the repair of double strand breaks (DSB) (Fig. 2a and Additional file 1: Figure S3A); Rad 51 is also inhibited, a protein with a major role in the homologous recombination repair (HRR [13, 27]). MPG protein is able to initiate the base excision repair (BER) mechanism that repairs alkylating bases [1, 39, 42] (Fig. 2a and Additional file 1: Figure S3A). The expression of these proteins is altered in refractory cancer cells as machinery of resistance to chemotherapy or radiotherapy [2, 44] .Interestingly, pH2Ax protein is also inhibited when tumor cells are infected, even after higher doses of irradiation (Fig. 2a and Additional file 1: Figure S3A). Phosphorylation of H2Ax marks a double strand break; therefore, pH2Ax inhibition could represent an advantage for the therapy. DNA repair machinery of infected tumor cells is unable to detect DNA damage caused by RT (Fig. 2b, c and Additional file 1: Figure S3B and S3C) and not repair it, increasing DNA damage that prompts in cell death.

Histological analyses showed that Delta-24-RGD combined with RT is able to replicate in pHGG and DIPG in vivo. Positive immuno-staining against viral proteins are found in tumors after irradiation in vivo (Fig. 2d).

Our results suggest that the synergistic antitumor effect that we observed in combination adenovirus/RT could be explained, at least in part, by the inhibition that the adenovirus exerts on the cell DNA damage repair machinery and the subsequent increase in DNA damage.

### Delta-24-RGD combined with RT enhanced the antitumor effect in murine DIPG cell lines

In addition, we wanted to elucidate how the immune response contributed to the synergistic effect of this combination.

**Fig. 3:**
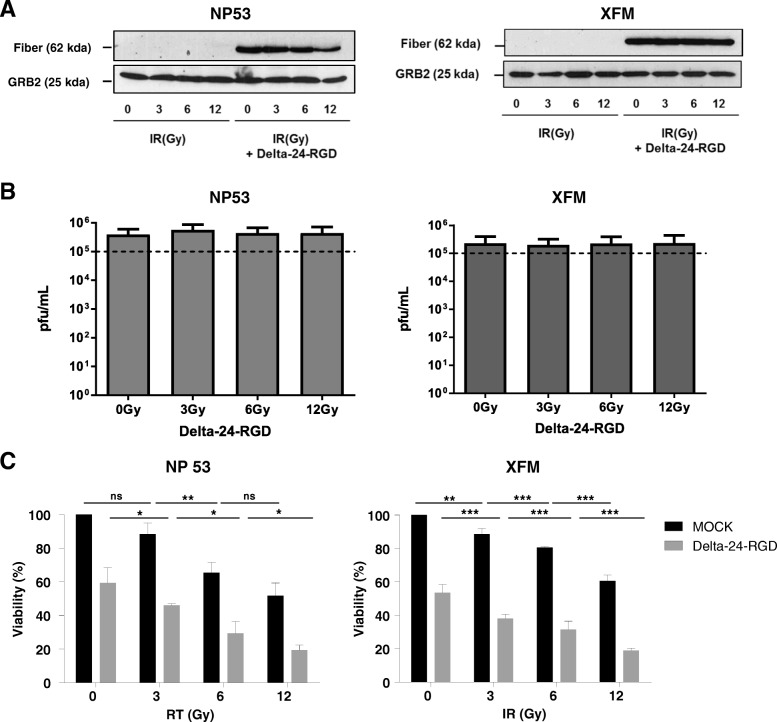
Combination of Delta-24-RGD/radiotherapy exerts a potent oncolytic effect in the NP53 and XFM murine DIPG cell lines. **a** Analyses of the expression of viral late protein fiber in murine cell lines 42 h after the indicated treatments by western blotting. **b** Quantification of Delta-24-RGD replication in the indicated cell lines. Viral titers were determined 3 days after infection with Delta-24-RGD (100 MOIs) and irradiation with either 3, 6 or 12 Gy. The viral titers were quantified using the anti-hexon staining-based method in 293 cells and expressed as plaque-forming units (pfu) per milliliter. The data are shown as the mean ± SD of three independent experiments. **c** Cell viability analyses of the combination treatment in DIPG murine cell lines. Cell viability was assessed 5 days after irradiation and/or viral infection using an automatic cell counter that measures cell viability (life, death and total cells) with the standard trypan blue reaction. Data are shown as the percentage (mean ± SD) of viability after irradiation at the indicated doses alone or also infected with Delta-24-RGD and analyzed with Two-tailed Student t-test

To this end, we evaluated the combination of Delta-24-RGD plus RT in the XFM and NP53 murine DIPG cell lines [3, 10]. Previously, we showed that these cell lines were semipermissive to Delta-24-RGD (Martinez-Velez et al., 2019 in review); therefore, these cell lines constitute a good model to analyze immune response to the viral infection. We observed that fiber protein, a late protein that is part of the adenoviral capsid, is robustly expressed after the highest dose of RT (Fig. 3a). At the functional level, we did not observe variations between viral titers obtained in non-irradiated versus irradiated infected cells (Fig. 3b). Importantly, treatment of these cell lines with RT alone was not capable of decreasing the viability more than 50% at the highest dose used (12 Gy), given that NP53 is the most sensitive cell line. The addition of Delta-24-RGD resulted in a significant decrease in cell viability, between 40 and 70%, compared with the single treatment (Fig. 3c).

Therefore, we can conclude that the combination of Delta-24-RGD/RT increases the cytotoxicity of a single treatment in DIPG murine cell lines in vitro.

### Delta-24-RGD /RT treatment triggers a potent immune response in a murine DIPG model

Since it has been described that anti-tumor immune effect mediated by local RT is more prominent after a previous immune activation [8, 32] we analyzed whether combined Delta-24-RGD/RT treatment resulted in a heightened immune response.

**Fig. 4:**
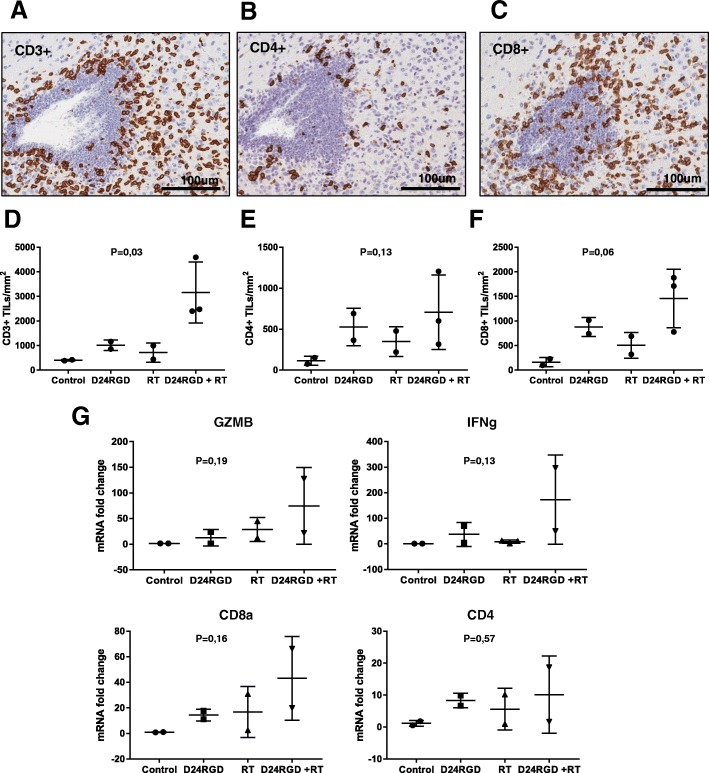
Administration of Delta-24-RGD in combination with radiotherapy heightens the immune infiltration in DIPG murine tumors. **a** Representative images of immune infiltration, such as perivascular cuffing, after Delta-24-RGD/RT treatment. The mouse brain stained against CD3 (**a**), CD4 (**b**) and CD8 (**c**). **d** Quantification of positive CD3+, **e** CD4+, and **f** CD8+ cell infiltration per mm^2^ of DIPG tumors. Graph showing the quantification of positive cells infiltrating the brain 15 days after the indicated treatments per mm^2^ (PBS, RT, Delta-24-RGD and Delta-24-RGD/RT; *n* = 2–3). Comparisons were analyzed with One-way ANOVA. **g** Quantification of Granzyme B, IFN gamma, CD8a and CD4 mRNA expression. The data shown represent mRNA expression in tumors treated with Delta-24-RGD, RT, and Delta-24-RGD/RT normalized to the average of PBS-tumors mRNA expression (*n* = 2). Data were analyzed with One-way ANOVA

Mice bearing NP53 DIPG tumors were administered a single injection of Delta-24-RGD followed 1 day later by RT. Histopathology examination of mice tumors treated with the combination showed an increase in tumor immune CD3 infiltration when compared with the single treatment. Moreover, we observed perivascular cuffing in mice brain treated with RT/Delta-24-RGD, indicating an immune cell recruitment triggered by the combination, mainly CD4+ and CD8+ (Figs. 4a, b, c and Additional file 1: Figure S4).

Quantification of CD3, CD4 and CD8 positive cells (Figs. 3d, e and f) showed a modest increase in lymphocyte infiltration in irradiated tumors (2.02, 3.06 and 3.08-fold, respectively); this infiltration was higher in tumors treated with Delta-24-RGD alone (2.46, 4.6 and 5.35-fold, respectively). Tumors treated with both agents showed a significant increase in the recruitment of immune populations (7.83, 6.2 and 8.89-fold) (CD3+, CD4+ and CD8+) (*P* = 0.032, *P* = 0.318 and *P* = 0.065), respectively. Evaluation of pro-inflammatory cytokines expression levels, such as Granzyme B (*P* = 0.19) and IFNg (*P* = 0.13), in addition to CD4 (*P* = 0.57) and CD8a (*P* = 0.16) expression levels, presented an increased trend in tumors treated with Delta-24-RGD and irradiation compared with single treatments (Fig. 3g), indicating that the combination stimulates the immune response in DIPG tumors. All together, these results demonstrate that combination of RT and Delta-24-RGD in vivo triggers a potent immune response that increases the immune recruitment at the tumor bed and the production of inflammatory cytokines that could mediate an antitumor immune response.

## Discussion

Previous works demonstrated that the oncolytic adenovirus Delta-24-RGD exerts a potent antitumor effect in pHGG and DIPG preclinical models. pHGG and specifically, DIPGs, are the most aggressive brain tumors with dismal outcomes and ineffective therapeutic options [38]. Clinical trials testing single agents as unique treatments have resulted in limited efficacy. The standard treatment for unresectable tumors is hypo fractionated RT [11], which improves the quality of life of these children transitorily, but in a few months, the tumors will relapse. Therefore, the valuation of the therapeutic benefit of the Delta-24-RGD/RT combination is extremely timely.

One of our findings was that the increase in cytotoxicity of Delta-24-RGD/RT in vitro and in vivo in pHGG and DIPG models could be mediated by the inhibition of the cellular DNA repair mechanisms resulting in an increase in DNA damage. Delta-24-RGD administration has been described to inhibit proteins involved in DNA repair to allow viral replication [29]. We observed the inhibition of proteins that play a main role in double strand break repair, such as Rad51, MPG and proteins that are part of the MRN complex, in pediatric glioma cells infected with Delta-24-RGD. The inhibition of the host cellular DNA repair machinery is sustained after irradiation, resulting in cellular machinery incapable of repairing the DNA damage and thus sensitizing cells to irradiation-induced cell death. Inhibition of Rad51 and MPG has been described to sensitize glioma cells to other agents [43], such as TMZ [2], a drug that is commonly used to treat brain tumors, opening new therapeutic combination options.

Recent evidence has shown that in addition to tumor growth delay mediated by DNA damage-induced cell death, RT also performs an immunostimulatory effect that is triggered by the activation of immune cells or by the modification of the tumor bed [8, 31]. The antitumor systemic effect found after local irradiation is defined as the abscopal effect and induces tumor recognition by immune cells [19]. Delta-24-RGD has been shown to unleash an immune response in a clinical trial phase I/II performed in recurrent adult gliomas [22].

We observed that Delta-24-RGD/RT increased the lymphocyte infiltration in DIPG tumors (including CD4+ and CD8+ cells), and the mRNA evaluation showed an increase in the expression of several cytokines. Viral administration stimulates immune infiltration, which that overcomes the immune “cold” status of these tumors [23]. It has been hypothesized that RT fails to develop an abscopal effect because of the active immunosuppression sustained by the tumor microenvironment. The immune system boost induced by Delta-24-RGD administration offers the immune activation that is necessary to stimulate the trafficking of immune specific effector cells into the tumor niche that RT needs to trigger the abscopal effect.

Therefore, Delta-24-RGD/RT administration could represent a promising therapeutic combination, considering the awakening of the immune system, which could increase the probability of developing a specific immune response against tumor cells that would translate to an effective antitumor effect.

Our results have supported the opening of a new clinical trial phase I/II in our institution to evaluate the safety and efficacy of Delta-24-RGD administration followed by RT in patients with newly diagnosed DIPG [37].

## Conclusion

In conclusion, Delta-24-RGD alone or in combination with RT is a promising therapy for DIPG and pHGG patients, and additional studies to understand the virus-induced immune response in patients could improve immune-virotherapy approaches to fight against these aggressive tumors.

## Additional file

Additional file 1: **Figure S1.** Combination of radiotherapy with the oncolytic virus Delta-24-RGD results in a potent oncolytic effect in the DIPG and pHGG cell lines. **Figure S2.** Delta-24-RGD in combination with radiotherapy shows a synergistic cytotoxic effect in the DIPG and pHGG cell lines in vitro. **Figure S3.** Delta-24-RGD downregulation of the cellular DNA damage repair machinery in the DIPG and pHGG cell lines. **Figure S4.** Administration of Delta-24-RGD in combination with radiotherapy heightens the immune infiltration in DIPG murine tumors. **Table S1.** Evaluation of dose-escalation RT administration to the mice pons. (PDF 1150 kb)
