# Supplementary material for: Delta-24-RGD combined with radiotherapy exerts a potent antitumor effect in diffuse intrinsic pontine glioma and pediatric high grade glioma models
Source: Acta Neuropathol Commun. 2019 Apr 29;7:64. doi: 10.1186/s40478-019-0714-6 (PMC6487528; doi:10.1186/s40478-019-0714-6)
Supplement: Supplementary file 1 — Figure S1. Combination of radiotherapy with the oncolytic virus Delta-24-RGD results in a potent oncolytic effect in the DIPG and pHGG cell lines. Figure S2. Delta-24-RGD in combination with radiotherapy shows a synergistic cytotoxic effect in the DIPG and pHGG cell lines in vitro. Figure S3. Delta-24-RGD downregulation of the cellular DNA damage repair machinery in the DIPG and pHGG cell lines. Figure S4. Administration of Delta-24-RGD in combination with radiotherapy heightens the immune infiltration in DIPG murine tumors. Table S1. Evaluation of dose-escalation RT administration to the mice pons. (PDF 1150 kb) [file 40478_2019_714_MOESM1_ESM.pdf]

Figure S1

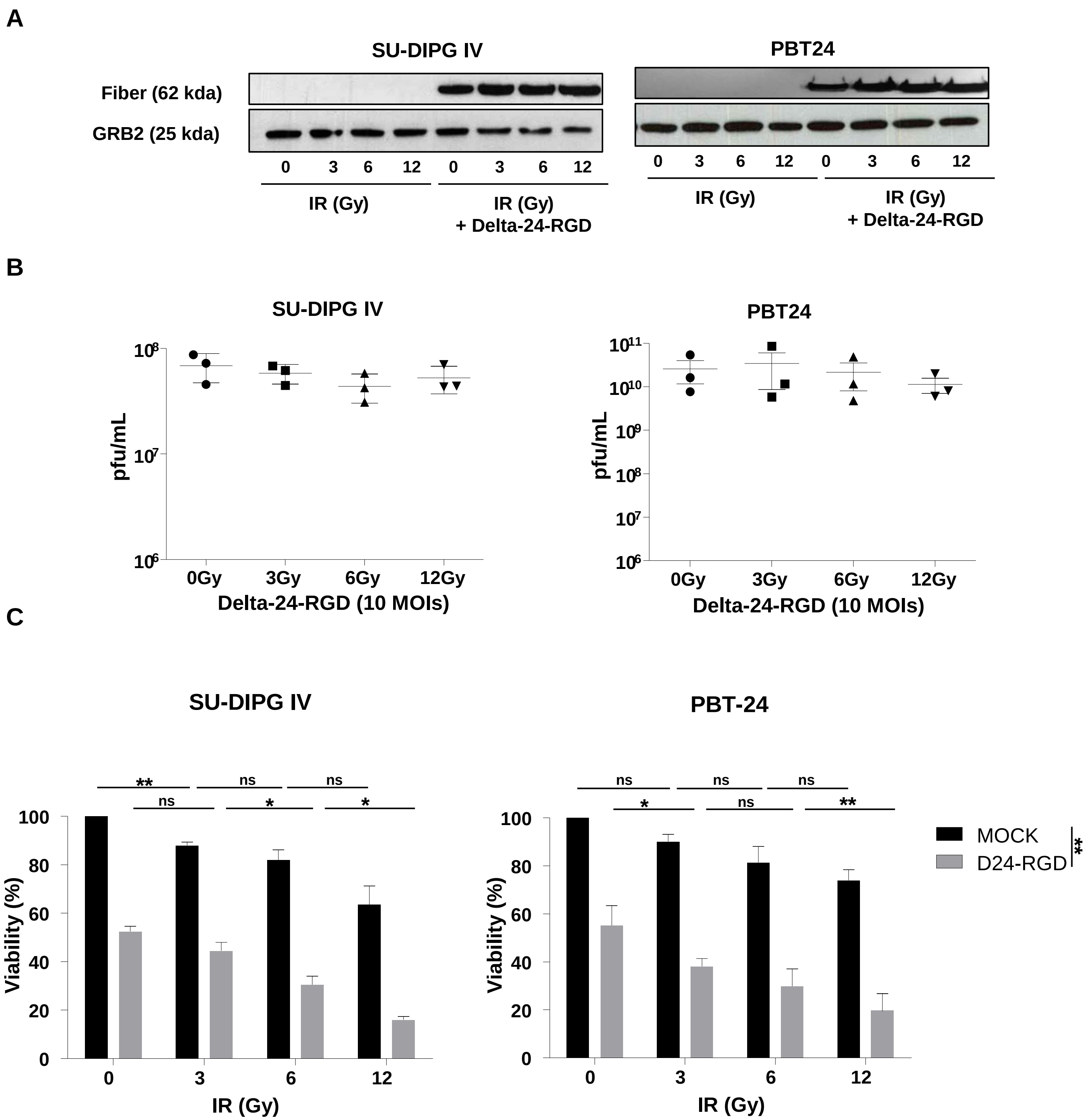

Figure S1. Combination of radiotherapy with the oncolytic virus Delta-24-RGD results in a potent oncolytic effect in the DIPG and pHGG cell lines. (**A**) Assessment by western blotting of viral protein expression in pediatric glioma cell lines, SU-DIPG IV and PBT-24, infected with Delta-24-RGD (10 MOIs) and irradiated with 0 Gy, 3 Gy, 6 Gy or 12 Gy. (**B**) Quantification of Delta-24-RGD replication in the indicated cell lines after viral infection alone or in combination with the indicated RT doses. The viral titers were determined 3 days after infection at an MOI of 10 by an anti-hexon staining-based method in 293 cells and expressed as plaque-forming units (pfu) per milliliter. Data are shown as the mean ± SD of three independent experiments. (**C**) Cell viability analyses of RT alone (at the indicated doses) or in combination with Delta-24-RGD. Cell viability was assessed 5 days after the indicated treatments using an automatic cell counter that measured cell viability (life, death and total cells) with the standard trypan blue reaction. Data are shown as the percentage (mean ± SD) of viability after irradiation alone or in combination with Delta-24-RGD relative to control cells (neither infected nor irradiated). Statistical analysis used was two-tailed Student t-test.

Figure S2

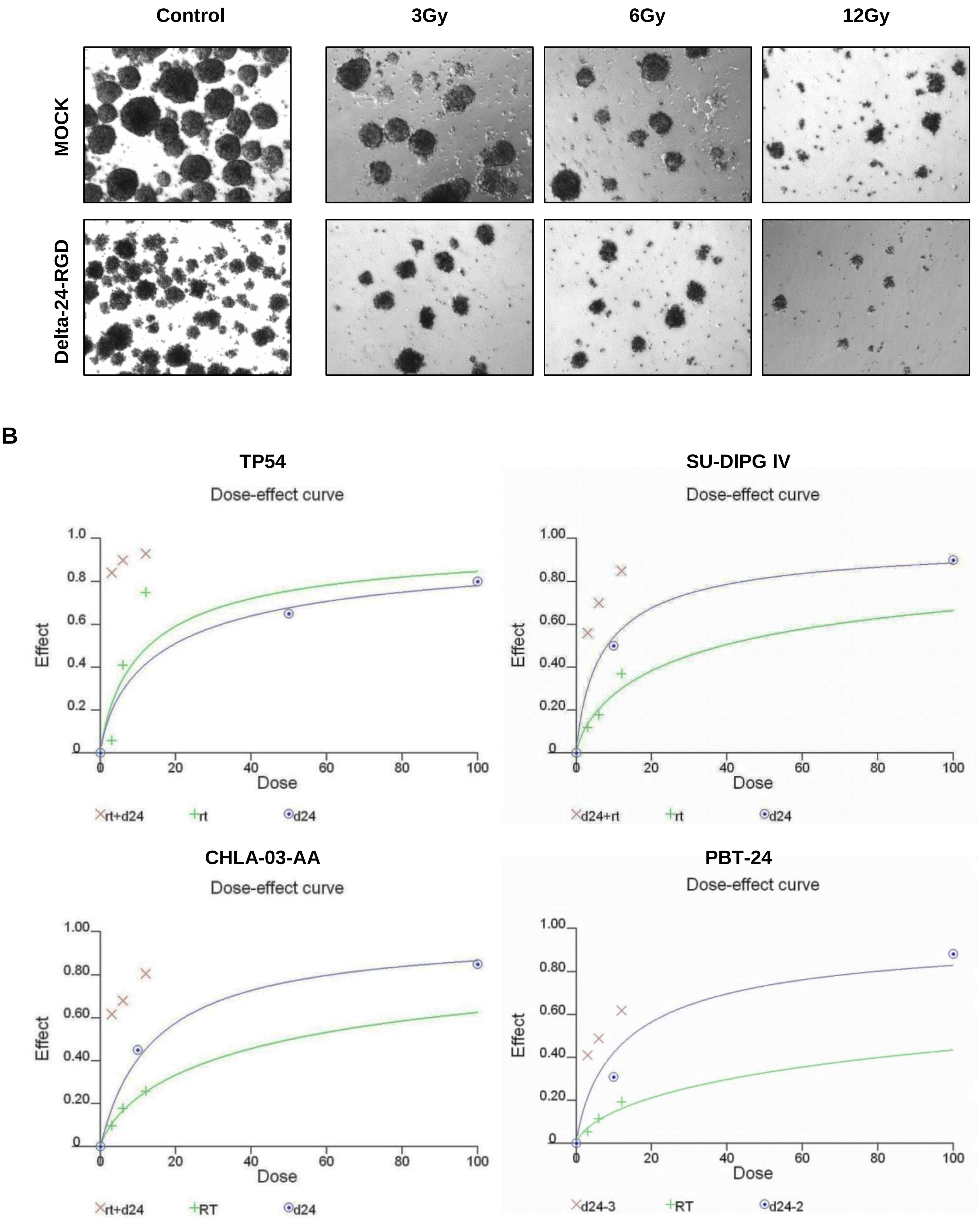

Figure S2. Delta-24-RGD in combination with radiotherapy shows a synergistic cytotoxic effect in the DIPG and pHGG cell lines in vitro. (**A**) Representative images of the cytopathic effect induced by the indicated treatments. (**B**) Representative graphs of combinatory index quantification. The curves represent the effect of Delta-24-RGD or RT or the combination of both treatments.

Figure S3

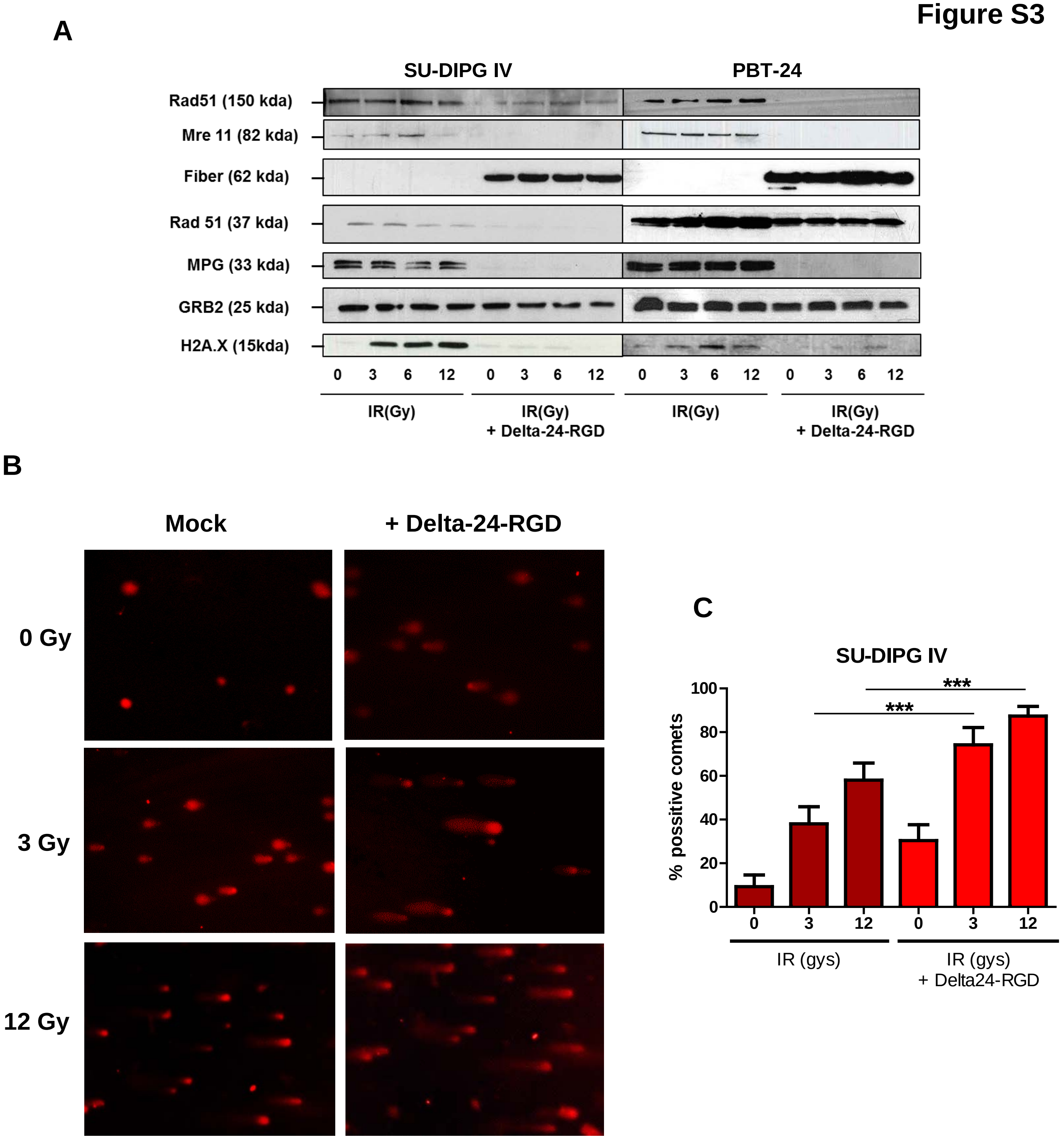

Figure S3. Delta-24-RGD downregulation of the cellular DNA damage repair machinery in the DIPG and pHGG cell lines. (**A**) Expression analyses by western blotting of the relevant proteins involved in the DNA damage response to RT in the DIPG (SU-DIPG IV) and pHGG (PBT-24) cell lines after the indicated treatments. Proteins levels were evaluated 72 hours after cells were treated. (**B**) Evaluation of DNA damage upon treatment with Delta-24-RGD and/or RT by the comet assay. SU-DIPG IV cells were administered the indicated treatments, and 72 hours, later the induction of comets were assessed. Representative photomicrographs of comets shown by cells after the indicated treatment (magnification, ×200). (**C**) Quantification of positive cells showing comets after the indicated treatment. Data are shown as the percentage of comet tails found per treatment percentage (n = 500 cells per treatment); *bars*, SD. All experiments were performed in triplicate and analyzed using the two-ways ANOVA with Bonferroni posttest.

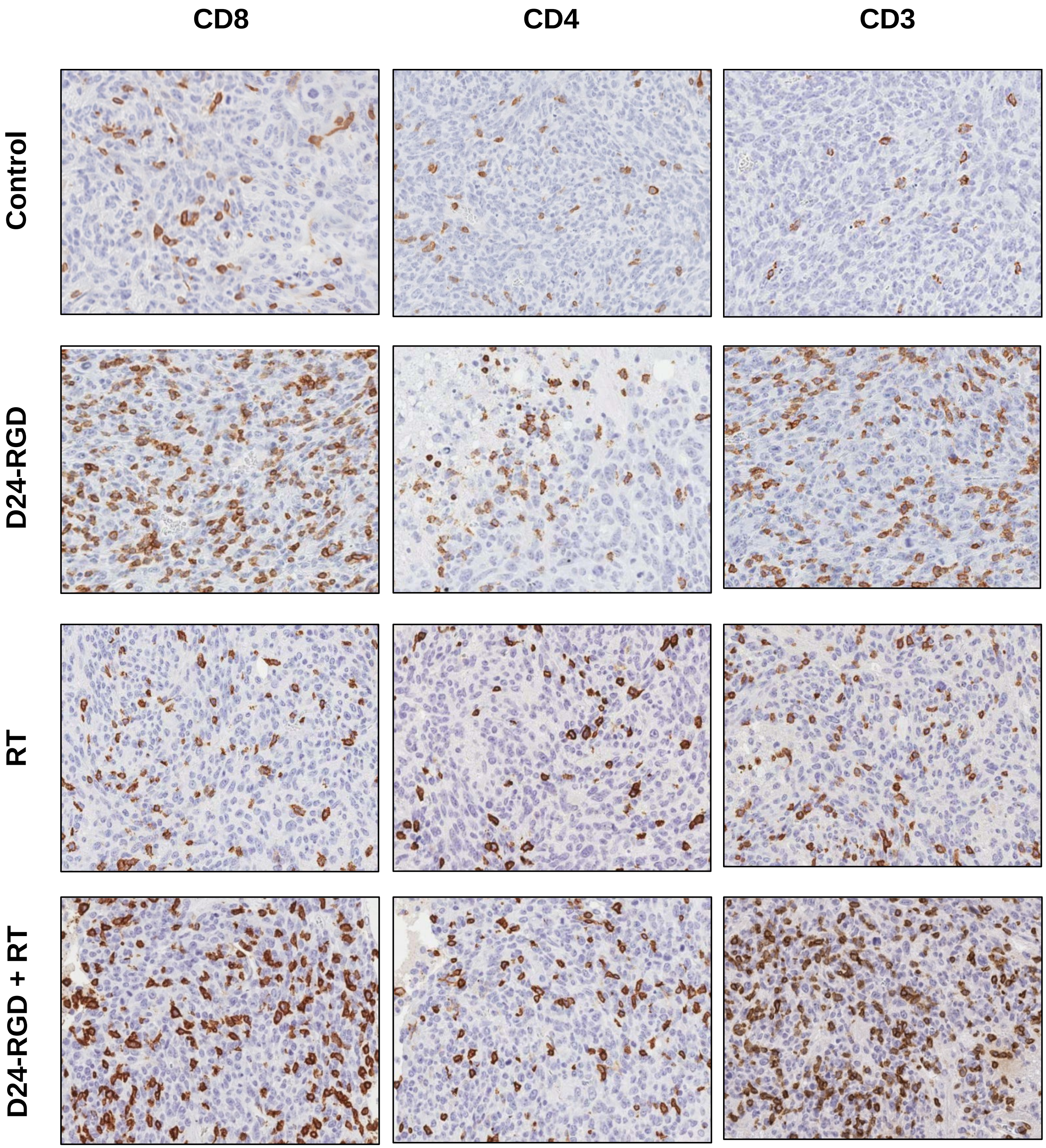

Figure S4. Administration of Delta-24-RGD in combination with radiotherapy heightens the immune infiltration in DIPG murine tumors. Representative images of immune infiltration in DIPG tumors no treated and after RT, Delta-24-RGD or Delta-24-RGD/RT treatment. The mouse brain stained against CD3, CD4 and CD8 as indicated. Scale bars = 100µm.

Table S1. Evaluation of dose-escalation RT administration to the mice pons.

**Treatment**	**Toxicity**
0 Gy (N=9)	0/10
4 Gy (N=10)	0/10
6 Gy (N=10)	0/10
8 Gy (N=10)	0/10
12 Gy (N=10)	0/10
